# Hemorrhagic Shock in Primary Hepatic Pregnancy: A Diagnostic and Surgical Challenge

**DOI:** 10.1155/cris/5393611

**Published:** 2025-08-19

**Authors:** Martin Manzaneda-Peralta, Jerson Morales-Rodriguez, Edith Ramos-Ocola, José Valdivia-López, Ylein Alvarez-Delgadillo, José Jiménez-Vera, Julio Fuenzalida-Valdivia, Karlo Terreros-Abril

**Affiliations:** ^1^Surgery Department, Hospital Carlos Alberto Seguin Escobedo, Arequipa, Peru; ^2^School of Medicine, National University of San Agustín, Arequipa, Peru; ^3^School of Human Medicine, Catholic University of Santa María, Arequipa, Peru

**Keywords:** case report, ectopic, hepatic, hypovolemia, pregnancy

## Abstract

**Introduction:** Primary hepatic ectopic pregnancy is rare; it has been reported to have an incidence of 1:15,000 per uterine pregnancy approximately. This study aims to determine the clinical presentation and treatment of hepatic ectopic pregnancy.

**Presentation of Case:** We present the case of a patient with no history of pregnancy who presented with abdominal pain refractory to treatment. With a human chorionic gonadotropin hormone (β-hCG) measure of 55,710 mIU/mL, an abdominal ultrasound that revealed the presence of a rounded image of 50 mm × 50 mm at the level of the right hepatic lobe and the complication of hypovolemic shock. Under the diagnosis of an abdominal ectopic pregnancy, the patient underwent surgery.

**Discussion:** Initially, an exploratory laparotomy was performed, which revealed the presence of bleeding, clots, and a gestational sac; subsequently, a wedge resection was done, and a Pringle maneuver and hepatic packing were performed, obtaining favorable results in the patient's case.

**Conclusion:** The diagnosis of primary hepatic ectopic pregnancy is made through β-hCG measurement and serial abdominal ultrasonography. Treatment can be pharmacological (methotrexate) or surgical, applying techniques such as the Pringle maneuver.

## 1. Introduction

An ectopic pregnancy is defined as one that occurs outside the uterine cavity. It is usually located in the fallopian tubes, ovaries, and nearby ligaments. However, locations such as liver or spleen are considered rare, approximately 1% of the total [1, 2]. It has an incidence of 1.4% and a mortality rate of 5.1/1000; the reason is that the risk of bleeding increases significantly in comparison with other presentations [3]. We present a case of a primary hepatic ectopic pregnancy, where its removal and the management of hepatic hemorrhage are of vital importance. This case report of ectopic hepatic pregnancy contributes to the limited available literature on this rare condition, emphasizing the diagnostic challenges and the importance of maintaining a high index of suspicion in patients with atypical symptoms.

## 2. Case Presentation

**Figure 1:**
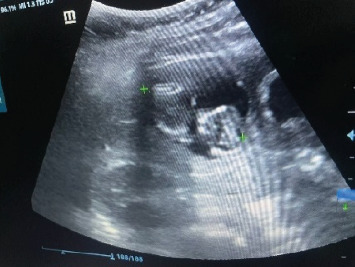
Abdominal ultrasound. A gestational sac and embryo approximately were visualized in the right hepatic lobe. Cardio-fetal beats were present, but the biometry was not measurable at that moment.

A 30-year-old woman with no history of previous pregnancy or diseases presented to the emergency department with abdominal pain refractory to treatment for 9 days, which was at first treated as gastritis. The following laboratory tests were performed: hemogram, C-reactive protein (CRP), platelets, and human chorionic gonadotropin hormone (β-hCG) (Table 1). The β-hCG dosage was measured at a value of 55,710 mUI/m. The abdominal ultrasound reported the presence of a rounded image of 50 mm × 50 mm with defined contours at the level of the right hepatic lobe, containing a fetus of approximately 13 weeks with active movements and heartbeat present (Figure 1). Moreover, free fluid of approximately 12 mL was also described. Other modalities of imageology tests were not applied.

Physical examination showed positive McBurney's and Blumberg's tender points and altered vital functions: BP 70/30 mmHg, CF 95 bpm, and a tendency to tachycardia. That is why she was transferred to the Shock Trauma unit, where she received intensive fluid therapy, with an inappropriate response. A few minutes later the patient was admitted to the emergency operating room with the diagnosis of ectopic pregnancy and hypovolemic shock due to internal bleeding to perform an exploratory laparotomy to control hemodynamic instability.

**Figure 2:**
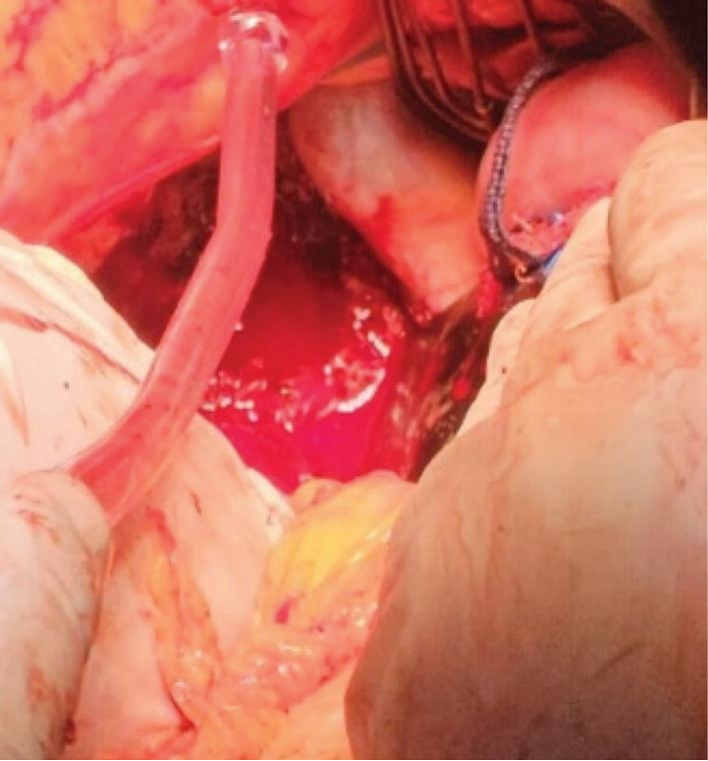
Site of the ectopic pregnancy found during the first surgical intervention.

**Figure 3:**
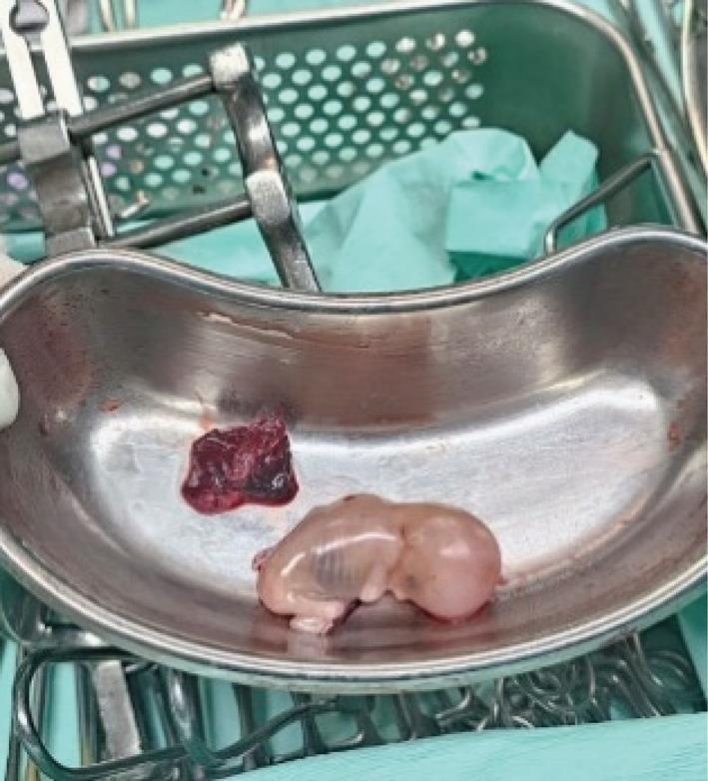
Fetal product extracted from the liver. Next to it, a small amount of trophoblastic tissue was evidenced.

Exploratory laparotomy revealed 1500 mL of blood and clots organized in the abdominal cavity. In the right hepatic lobe (segment VI), a gestational sac was visualized, with embryonic remains and placenta (Figures 2, 3). A wedge resection, Pringle maneuver, and hepatic packing were performed. The gynecological report described the uterus, tubes, and ovaries preserved without any active bleeding point. Right after, the patient was transferred to the ICU for postoperative management and support with mechanical ventilation and vasopressors.

On the first postoperative day, hemoglobin decreased to 6.9 g/dL. This was despite the transfusion of blood products. A new ultrasound showed free fluid at 100 cc with signs suggesting hepatic hematoma, so she was readmitted to the operating room for the review of hemostasis. In this intervention, serosanguineous drainage was evidenced in 150 mL, and hepatic sutures were in good condition with no signs of bleeding. The hepatic compresses were changed, and a laminar drain was placed in the Douglas pouch. Once the service of general surgery completed the procedure, the patient returned to the ICU.

**Figure 4:**
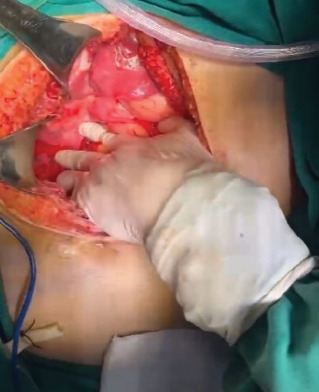
Last intervention. On the fourth postoperative day, the hepatic packing was removed. At this stage, no bleeding or significant lesions were seen.

The hepatic packing was removed on the fourth postoperative day without complications (Figure 4). After being stable, the patient was transferred to the hospitalization service on the sixth postoperative day. The laboratory results showed β-hCG values of 292 mIU/mL (Table 1). Finally, the patient was discharged, and the gynecology service prescribed β-hCG monitoring.

## 3. Discussion

Primary hepatic ectopic pregnancy is rare and extremely dangerous, with an incidence of 1:15,000 per uterine pregnancy [4]. Symptoms, such as pain refractory treatment or free fluid in the abdominal cavity have been reported [5, 6]. The possible pathophysiology involves intestinal and respiratory movements contiguous to the uterus that mobilize the fertilized ovum to the abdominal cavity, carried to the liver by the bloodstream [5]. The egg attaches to the fibrous capsule of the liver, the gestational sac develops, and the chorion infiltrates the liver, mainly appearing in the right lobe of the liver [7].

The clinical presentation of an abdominal ectopic pregnancy is usually asymptomatic unless persistent abdominal pain, rebound pain, muscle tension, bleeding, hemodynamic instability, and free fluid in the peritoneal cavity are found [6, 8, 9]. The levels of β-hCG may be elevated in female patients of childbearing age with a history of amenorrhea, even if there is no bleeding [3, 4, 9]. In the present case, the symptoms of the patient were at first considered as gastritis.

Currently, there are no guidelines from major institutions for the diagnosis of hepatic pregnancy. Literature indicates that diagnosis is based on a comprehensive assessment incorporating clinical suspicion (e.g., abdominal pain, amenorrhea, signs of intra-abdominal bleeding), elevated serum β-hCG, and imaging—primarily ultrasound [6, 9, 10]. If necessary, additional imaging studies can be performed, such as a CT scan or MRI, to localize the gestational sac to the hepatic surface [6]. However, these are recommendations derived from case reports, case series, and professional opinion rather than formal guidelines. In our case the blood test and imaging-primarily ultrasound study gave the main findings for the diagnosis.

Differential diagnosis requires certain considerations, mainly the clinical presentation, so in our case it presents as acute abdominal pain, making it important to establish other possible causes apart from ectopic pregnancy, such as appendicitis, early pregnancy loss, ovarian torsion, pelvic inflammatory disease, subchorionic hemorrhage in viable intrauterine pregnancy, trauma, urinary calculi, making it possible to guide the diagnosis according to the clinical presentation and support with auxiliary tests [10].

Therapeutic options include close monitoring of the patient, pharmacological therapy, and surgical intervention [5, 10]. In this case, surgical procedure was a priority over pharmacological therapy (e.g., methotrexate) to control the hemorrhage and limit hemodynamic instability.

Methotrexate is an effective pharmacological therapeutic option because it reduces DNA biosynthesis by inhibiting the enzyme dihydrofolate reductase and placental trophoblast division [9]. Its effectiveness decreases as gestation is more advanced and chorionic hormone levels are higher (>5000 IU/mL); its use is recommended under the following conditions: absence of hemodynamic instability, gestational sac less than 3.5 cm, and absence of other contraindications [10]. Its use postoperatively is not well described for hepatic pregnancy, but it showed favorable results in some patients.

The Pringle maneuver can control hepatic flow when resection or hepatectomy is performed, reducing intraoperative bleeding, the number of blood products transfused, and surgery time [11]. On the other hand, complete extraction is recommended only in cases where it is safe. In some cases, the possibility of ligating the cord as close as possible to the placenta and leaving it as an ideal option is considered [12]. In our case it was preferred to extract the placenta and even hepatic tissue for enhancing hepatic cicatrization.

## 4. Conclusion

Hepatic ectopic pregnancy is rare and requires early diagnosis to avoid fatal consequences. Its clinical presentation is usually asymptomatic. However, some patients may manifest amenorrhea and abdominal discomfort. For diagnosis, β-hCG measurement and serial abdominal ultrasonography should be performed.

Treatment with methotrexate is a valid therapeutic option if the conditions of use are met. On the other hand, surgery using the Pringle maneuver has shown promising results in controlling hepatic flow during hepatectomy, especially in the context of hemodynamic instability. Further comparative studies should be performed to demonstrate the efficacy of this technique.

The lack of formalized, consensus-based guidelines can make it difficult for the diagnostic approach and treatment of a patient with a hepatic or abdominal pregnancy. Major societies may consider addressing this pathology in future guidelines to provide guidance to obtain an adequate diagnostic and treatment approach for this type of patient.

## Figures and Tables

**Table 1:** Laboratory results by chronological order.

	First hours after the 1st surgery (24/06/12)	2 days after the 1st surgery (24/06/14)	First hours after the 2nd surgery (24/06/14)	1 day after the 2nd surgery (24/06/15)	2 days after the 2nd surgery (24/06/16)	Random control taken 11 days after the 2nd surgery (24/06/25)
Leukocytes (10^3^/µL)	10.540	14.570	19.430	12.980	19.140	7.400
Band cells (%)	0	2	13	4	20	0
Hemoglobine (g/dL)	12.8	9.6	7.9	6.8	10.2	11.4
Platelets (10^3^/µL)	305	266	218	193	169	665
PCR (mg/dL)	0.739	1.26	—	2.8	—	4.8
β-hCG (mUI/mL)	—	55,710	—	—	2928	292

## Data Availability

Data sharing is not applicable to this article as no datasets were generated or analyzed during the current study.
